# Immunogenicity of adjuvanted versus high-dose inactivated influenza vaccines in older adults: a randomized clinical trial

**DOI:** 10.1186/s12979-023-00355-7

**Published:** 2023-07-01

**Authors:** Kenneth E. Schmader, Christine K. Liu, Brendan Flannery, Wes Rountree, Heidi Auerbach, Elizabeth D. Barnett, Elizabeth P. Schlaudecker, Christopher A. Todd, Marek Poniewierski, Mary A. Staat, Theresa Harrington, Rongxia Li, Karen R. Broder, Emmanuel B. Walter

**Affiliations:** 1 Division of Geriatrics, Department of Medicine and Center for the Study of Aging, Duke University School of Medicine, Durham, NC USA; 2 Geriatric Research Education and Clinical Center (GRECC), Durham VA Health Care System, Box 3003, Durham, NC 27710 USA; 3 Section of Geriatrics, Division of Primary Care and Population Health, Stanford University, Stanford, CA USA; 4 Geriatric Research and Education Clinical Center (GRECC), Palo Alto Veterans Affairs Health Care System, Palo Alto, CA USA; 5 Geriatrics Section, Department of Medicine, School of Medicine and Boston Medical Center, Boston University, Chobanian & Avedisian, Boston, MA USA; 6 Infuenza Division, National Center for Immunization and Respiratory Diseases, Centers for Disease Control and Prevention (CDC), Atlanta, GA USA; 7 Duke Human Vaccine Institute, Duke University School of Medicine, Durham, NC USA; 8 Department of Pediatrics, Section of Pediatric Infectious Diseases, School of Medicine and Boston Medical Center, Boston University, Chobanian & Avedisian, Boston, MD USA; 9 Department of Pediatrics Division of Infectious Diseases, University of Cincinnati College of Medicine and Cincinnati Children’s Hospital and Medical Center, Cincinnati, OH USA; 10 Immunization Safety Office, Centers for Disease Control and Prevention (CDC), Atlanta, GA USA; 11 Department of Pediatrics, Duke University School of Medicine, Durham, NC USA

**Keywords:** Influenza vaccine, Immunogenicity, Aged, Adjuvanted influenza vaccine, High dose influenza vaccine

## Abstract

**Background:**

Adjuvanted inactivated influenza vaccine (aIIV) and high-dose inactivated influenza vaccine (HD-IIV) are U.S.-licensed for adults aged ≥ 65 years. This study compared serum hemagglutination inhibition (HAI) antibody titers for the A(H3N2) and A(H1N1)pdm09 and B strains after trivalent aIIV3 and trivalent HD-IIV3 in an older adult population.

**Results:**

The immunogenicity population included 342 participants who received aIIV3 and 338 participants who received HD-IIV3. The proportion of participants that seroconverted to A(H3N2) vaccine strains after allV3 (112 participants [32.8%]) was inferior to the proportion of participants that seroconverted after HD-IIV3 (130 participants [38.5%]) at day 29 after vaccination (difference, − 5.8%; 95%CI, − 12.9% to 1.4%). There were no significant differences between the vaccine groups in percent seroconversion to A(H1N1)pdm09 or B vaccine strains, in percent seropositivity for any of the strains, or in post-vaccination GMT for the A(H1N1)pdm09 strain. The GMTs for the post-vaccination A(H3N2) and B strains were higher after HD-IIV than after aIIV3.

**Conclusions:**

Overall immune responses were similar after aIIV3 and HD-IIV3. For the primary outcome, the aIIV3 seroconversion rate for H3N2 did not meet noninferiority criteria compared with HD-IIV3, but the HD-IIV3 seroconversion rate was not statistically superior to the aIIV3 seroconversion rate.

**Trial registration:**

ClinicalTrials.gov Identifier: NCT03183908.

**Supplementary Information:**

The online version contains supplementary material available at 10.1186/s12979-023-00355-7.

## Background

The Centers for Disease Control and Prevention (CDC) Advisory Committee on Immunization Practices (ACIP) recommends annual vaccination with any U.S.-licensed, age-appropriate, influenza vaccine [9]. In the U.S., two influenza vaccines licensed and recommended only for persons aged ≥ 65 years are quadrivalent high-dose inactivated influenza vaccine [HD-IIV4 (Fluzone® High-Dose Quadrivalent) (Package Insert)] [5] and quadrivalent inactivated adjuvanted influenza vaccine [aIIV4 (Fluad® Quadrivalent) (Package Insert)] [6]. At the time this study was enrolling participants, quadrivalent formulations were not yet licensed; trivalent high-dose inactivated influenza vaccine [HD-IIV3 (Fluzone® High-Dose) (Package Insert)] [7] and trivalent adjuvanted inactivated influenza vaccine [aIIV3 (Fluad®) (Package Insert)] [8] were the only U.S. influenza vaccines approved exclusively for use in persons aged ≥ 65 years.

Seasonal influenza vaccines are less immunogenic in older adults compared to younger adults [3]. To improve the immunogenicity of influenza vaccines in older adults, vaccines have been developed which contain an increased the dose of hemagglutinin antigen (HD-IIV) or which contain an adjuvant (aIIV). [12, 16]. HD-IIV has four times the dose of each hemagglutinin antigen compared to standard-dose IIV (SD-IIV) [7]. Compared with SD-IIV, HD-IIV is significantly more immunogenic for the influenza A strains and noninferior for the B strains [7]. aIIV contains the MF59 adjuvant which is a squalene-based, oil-in-water emulsion [16]. Compared with SD-IIV, the MF-59 adjuvanted influenza vaccine may produce higher antibody responses and broadens the immune response to circulating influenza viruses in older adults [16]. Noninferiority of aIIV3 compared with SD-IIV3 was demonstrated for all three vaccine strains based on pre-defined thresholds for seroconversion rate differences and geometric mean titer ratios (FLUAD Package Insert).

There are few data on the comparative immunogenicity of aIIV and IIV-HD in older adults. To better understand the comparative immunogenicity of these vaccines, we conducted a randomized clinical trial in older adults. The primary objective of this study was to compare seroconversion based on serum hemagglutination inhibition (HAI) antibody titer for the A (H3N2) strain after receipt of either aIIV3 or HD-IIV3 in an older adult population. A (H3N2) was chosen because this subtype is the leading cause of influenza illness and mortality in older adults [10]. We hypothesized that the seroconversion rate for the A(H3N2) strain after aIIV3 would be noninferior to HD-IIV3. Secondary objectives were to compare seroconversion rates for A(H1N1)pdm09 and B vaccine strains, and post-vaccination HAI antibody geometric mean titers (GMT) and seropositivity for each of the three influenza vaccine strains after aIIV3 or HD-IIV3 by season and age-group in the full study population. We explored associations between immunogenicity and reactogenicity, as well as between immunogenicity and age-group, sex, race, and statin use in participants receiving aIIV3 and IIV3-HD. Antibody titers for influenza vaccine antigens 6 months after vaccination were evaluated in a subset of participants.

## Results

### Study participants

**Fig. 1:**
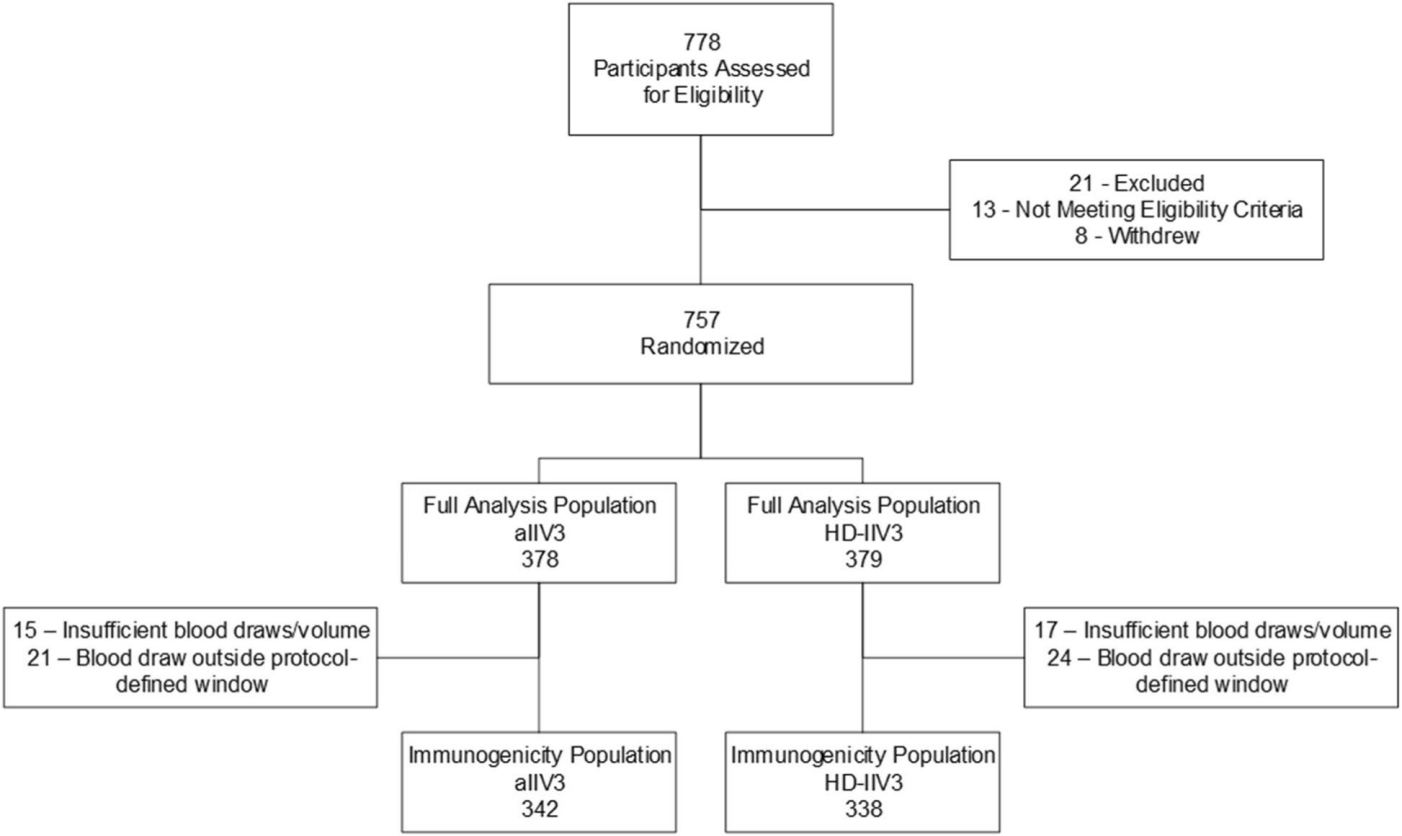
Randomization and Patient Flow in the Immunogenicity Study Comparing Trivalent Adjuvanted Inactivated Influenza Vaccine (aIIV3) vs. Trivalent High-Dose Inactivated Influenza Vaccine (HD-IIV3). The Full Analysis Population consisted of all participants who were randomized and vaccinated. The Immunogenicity Population consisted of all participants who were randomized, vaccinated and had a sufficient blood draw within the protocol defined time window

We assessed 778 participants for eligibility during two influenza seasons. Twenty-one participants were excluded (8 participants withdrew prior to randomization and 13 participants did not meet eligibility criteria), yielding 757 randomized participants; 378 received aIIV3 and 379 received HD-IIV3 in the full analysis population (Fig. 1). Of the 378 participants who received aIIV3, 15 had insufficient blood draws and 21 had blood draws outside the protocol-defined window, leaving 342 participants in the immunogenicity population. Of the 379 participants who received HD-IIV3, 17 had insufficient blood draws and 24 had blood draws outside the protocol-defined window, leaving 338 participants in the immunogenicity population. The baseline demographic and clinical characteristics of participants in the immunogenicity population were similar between the 2 study groups (Table 1). We randomized 279 participants in the 2017–2018 influenza season and 478 participants in the 2018–2019 influenza season. There were 45 participants in the aIIV3 group and 47 participants in the HD-IIV3 group in the subset of individuals with immune measures at 181 days post vaccination.

**Table 1:** Baseline demographic and clinical characteristics of the immunogenicity population for 2017–2018 and 2018–2019 seasons

	Patients vaccinated, No. (%)	
Characteristic	aIIV3^a^ (*n* = 342)	HD-IIV3^b^ (*n* = 338)
Study Site		
Duke University Medical Center	191 (55.8%)	186 (55%)
Boston Medical Center	109 (31.9%)	110 (32.5%)
Cincinnati Children’s Hospital Medical Center	42 (12.3%)	42 (12.4%)
Influenza Season of Enrollment		
2017–2018	123 (36%)	122 (36.1%)
2018–2019	219 (64%)	216 (63.9%)
Age, median (range), y	72 (65–96)	72 (65–97)
Age group, y		
65–79	269 (78.7%)	261 (77.2%)
≥ 80	73 (21.3%)	77 (22.8%)
Female	191 (55.8%)	183 (54.1%)
Race		
White only	258 (75.4%)	274 (81.1%)
Black only	62 (18.1%)	50 (14.8%)
Other	22 (6.4%)	14 (4.1%)
Hispanic ethnicity	7 (2%)	1 (0.3%)
Education, some college or higher	300 (87.7%)	296 (87.8%)
Employment		
Employed	24 (7%)	23 (6.8%)
Retired	271 (79.2%)	266 (78.7%)
None	14 (4.1%)	14 (4.1%)
Living Alone	137 (40.1%)	131 (38.8%)
Cardiovascular and respiratory disorders		
Atrial fibrillation	28 (7.4%)	18 (4.7%)
Coronary artery disease	23 (6.1%)	22 (5.8%)
Heart failure	20 (5.3%)	9 (2.4%)
Hyperlipidemia	124 (32.8%)	133 (35.1%)
Hypertension	76 (20.1%)	63 (16.6%)
Valvular heart disease	14 (3.7%)	10 (2.6%)
Asthma	7 (1.9%)	8 (2.1%)
Chronic obstructive pulmonary disease	6 (1.6%)	1 (0.3%)
Other common conditions		
Arthritis	62 (16.4%)	64 (16.9%)
Depression	45 (11.9%)	42 (11.1%)
Diabetes	19 (5.0%)	24 (6.3%)
Gastroesophageal reflux disease	35 (9.3%)	20 (5.3%)
Hearing loss	8 (2.1%)	13 (3.4%)
Hypothyroidism	35 (9.3%)	28 (7.4%)
Statin Use	168 (49.1%)	157 (46.4%)
Received influenza vaccine in the previous season	323 (94.4%)	324 (95.9%)

^a^aIIV3: Trivalent adjuvanted inactivated influenza vaccine

^b^HD-IIV3: Trivalent high-dose inactivated influenza vaccine

### Primary outcome: seroconversion rate A(H3N2)

The proportion of participants that seroconverted after allV3 (112 participants [32.7%]) was inferior to the proportion of participants that seroconverted after HD-IIV3 (130 participants [38.5%]) at day 29 after vaccination (Table 2). The null hypothesis of inferiority for the aIIV3 seroconversion rate to the HD-IIV3 seroconversion rate was not rejected (*p* = 0.12) with a point estimate of the difference at -5.79% and associated 95% CI (-12.91%, 1.41%). Therefore, we cannot claim that the aIIV3 seroconversion rate is noninferior to HD-IIV3, but there is no statistical evidence that the HD-IIV3 seroconversion rate is superior since the confidence interval of the difference crosses zero.

**Table 2:** Hemagglutination Inhibition (HAI) antibody titers after trivalent adjuvanted inactivated influenza vaccine (aIIV3) and trivalent high-dose inactivated influenza vaccine (HD-IIV3) for each influenza vaccine strain by season(s) 2017–2018 and 2018–2019 influenza seasons. 2017–2018 and 2018–1019 influenza seasons

			aIIV3			HD-IIV3			aIIV3–HD-IIV3
Strain		Time	N	Value	95% CI	N	Value	95% CI	Difference 95% CI
2017–2018 and 2018–1019 influenza seasons									
H1N1	GMT^c^	Day 1^f^	342	51.8	(44.5, 60.3)	338	50.8	(43.4, 59.4)	0.01 (-0.09, 0.10)
		Day 29	342	100.0	(86.6, 115.6)	338	98.0	(84.6, 113.6)	0.01 (-0.08, 0.10)
	%SP^d^	Day 1	238	69.6	(64.4, 74.4)	226	66.9	(61.6, 71.8)	2.73 (-4.27, 9.72)
		Day 29	285	83.3	(79.0, 87.1)	282	83.4	(79.0, 87.2)	-0.10 (-5.69, 5.50)
	%SC^e^	Day 29	96	28.1	(23.4, 33.2)	90	26.6	(22.0, 31.7)	1.44 (-5.26, 8.14)
H3N2	GMT	Day 1	342	63.4	(52.8, 76.1)	338	58.5	(48.4, 70.7)	0.03 (-0.08, 0.15)
		Day 29	342	141.7	(123.2, 162.9)	338	177.3	(152.7, 205.9)	-0.10 (-0.19, -0.01)
	%SP	Day 1	228	66.7	(61.4, 71.6)	222	65.7	(60.4, 70.7)	0.99 (-6.13, 8.10)
		Day 29	307	89.8	(86.1, 92.7)	299	88.5	(84.6, 91.6)	1.30 (-3.38, 5.99)
	%SC	Day 29	112	32.7	(27.8, 38.0)	130	38.5	(33.2, 43.9)	-5.71 (-12.90, 1.47)
Influenza B	GMT	Day 1	342	13.4	(11.9, 15.0)	338	14.7	(12.9, 16.7)	-0.04 (-0.11, 0.03)
		Day 29	342	21.6	(18.5, 25.2)	338	27.5	(23.5, 32.3)	-0.11 (-0.20, -0.01)
	%SP	Day 1	84	24.6	(20.1, 29.5)	91	26.9	(22.3, 32.0)	-2.36 (-8.93, 4.21)
		Day 29	151	44.2	(38.8, 49.6)	165	48.8	(43.4, 54.3)	-4.66 (-12.15, 2.83)
	%SC	Day 29	64	18.7	(14.7, 23.3)	79	23.4	(19.0, 28.3)	-4.66 (-10.78, 1.46)
2017–2018 influenza season^a^									
H1N1	GMT^c^	Day 1^f^	123	29.2	(23.7, 36.0)	122	29.3	(23.6, 36.5)	-0.00 (-0.13, 0.13)
		Day 29	123	88.7	(68.5, 114.8)	122	88.6	(67.6, 116.2)	0.00 (-0.16, 0.16)
	%SP^d^	Day 1	61	49.6	(40.5, 58.7)	59	48.4	(39.2, 57.5)	1.23 (-11.29, 13.75)
		Day 29	96	78.0	(69.7, 84.9)	100	82.0	(74.0, 88.2)	-3.92 (-13.92, 6.08)
	%SC^e^	Day 29	47	38.2	(29.6, 47.4)	52	42.6	(33.7, 51.9)	-4.41 (-16.69, 7.87)
H3N2	GMT	Day 1	123	69.7	(53.2, 91.3)	122	78.7	(59.5, 104)	-0.05 (-0.22, 0.11)
		Day 29	123	228.2	(178.7, 291.4)	122	281.6	(213.6, 371.3)	-0.09 (-0.25, 0.07)
	%SP	Day 1	84	68.3	(59.3, 76.3)	87	71.3	(62.4, 79.1)	-3.02 (-14.51, 8.47)
		Day 29	117	95.1	(89.7, 98.0)	108	88.5	(81.5, 93.4)	6.60 (-0.22, 13.41)
	%SC	Day 29	55	44.7	(35.7, 53.9)	54	44.3	(35.3, 53.5)	0.45 (-11.99, 12.90)
Influenza B	GMT	Day 1	123	19.2	(15.9, 23.3)	122	18.9	(15.3, 23.4)	0.01 (-0.12, 0.13)
		Day 29	123	27.3	(21.5, 34.6)	122	37.6	(29.3, 48.3)	-0.14 (-0.29, 0.01)
	%SP	Day 1	43	35.0	(26.6, 44.1)	44	36.1	(27.6, 45.2)	-1.11 (-13.09, 10.88)
		Day 29	62	50.4	(41.2, 59.5)	74	60.7	(51.4, 69.3)	-10.25 (-22.63, 2.13)
	%SC	Day 29	19	15.4	(9.6, 23.1)	29	23.8	(16.5, 32.3)	-8.32 (-18.22, 1.57)
2018–2019 influenza season^b^									
H1N1	GMT^c^	Day 1^f^	219	71.5	(58.9, 86.8)	216	69.2	(56.5, 84.8)	0.01 (-0.11, 0.14)
		Day 29	219	107.0	(89.9, 127.5)	216	103.7	(87.1, 123.5)	0.01 (-0.09, 0.12)
	%SP^d^	Day 1	177	80.8	(75.0, 85.8)	167	77.3	(71.1, 82.7)	3.51 (-4.13, 11.15)
		Day 29	189	86.3	(81.0, 90.5)	182	84.3	(78.7, 88.8)	2.04 (-4.62, 8.70)
	%SC^e^	Day 29	49	22.4	(17.0, 28.5)	38	17.6	(12.8, 23.3)	4.78 (-2.72, 12.28)
H3N2	GMT	Day 1	219	60.1	(47.1, 76.7)	216	49.5	(38.6, 63.5)	0.08 (-0.07, 0.23)
		Day 29	219	108.4	(92.3, 127.3)	216	136.5	(115.6, 161.2)	-0.10 (-0.20, -0.00)
	%SP	Day 1	144	65.8	(59.1, 72.0)	135	62.5	(55.7, 68.9)	3.25 (-5.76, 12.26)
		Day 29	190	86.8	(81.5, 90.9)	191	88.4	(83.4, 92.3)	-1.67 (-7.86, 4.53)
	%SC	Day 29	57	26.0	(20.3, 32.4)	76	35.2	(28.8, 41.9)	-9.16 (-17.78, -0.54)
Influenza B	GMT	Day 1	219	10.9	(9.5, 12.5)	216	12.8	(10.9, 15.0)	-0.07 (-0.16, 0.02)
		Day 29	219	19.0	(15.6, 23.1)	216	23.1	(18.9, 28.3)	-0.09 (-0.21, 0.04)
	%SP	Day 1	41	18.7	(13.8, 24.5)	47	21.8	(16.4, 27.9)	-3.04 (-10.59, 4.51)
		Day 29	89	40.6	(34.1, 47.5)	91	42.1	(35.5, 49.0)	-1.49 (-10.75, 7.77)
	%SC	Day 29	45	20.5	(15.4, 26.5)	50	23.1	(17.7, 29.3)	-2.60 (-10.36, 5.16)

^a^Virus strains H1N1: A/Michigan/45/2015 (H1N1) pdm09-like virus; H3N2: A/Hong Kong /4801/2014 (H3N2)-like virus; B: B/Brisbane/60/2008-like virus (Victoria lineage)

^b^Virus strains H1N1: A/Michigan/45/2015 (H1N1)pdm09-like virus; H3N2: A/Singapore/INFIMH-16–0019/2016 A(H3N2)-like virus; B: B/Colorado/06/2017-like virus (Victoria lineage)

^c^GMT: Geometric mean titers

^d^%SP: Percent Seropositive, defined as HAI titer ≥ 1:40

^e^%SC: Percent Seroconversion, defined as an HAI titer ≥ 1:40 at day 29 post-vaccination if the baseline pre-vaccination titer is < 1:10 or a minimum four-fold rise in HAI titer if the baseline pre-vaccination titer is ≥ 1:10

^f^Day 1: Baseline blood draw before vaccination; Day 29 blood draw on 29 ± 7 days post-vaccination

### Secondary outcomes

For the H3N2 strain, there were no significant differences in the percent seropositive between the aIIV3 and HD-IIV3 groups for all ages or for either age group 65 to 79 years and 80 years and older (Tables 2 and 3). There was no difference (aIIV3 minus HD-IIV3), in Day 29 GMTs for the ≥ 80-year group but there was a difference for all ages (GMT aIIV3, 141.7 vs. IIV-HD, 177.3; difference, -0.10 (95% CI, -0.19, -0.01)) and the 65–79-year age group (GMT aIIV3, 145.3 vs. IIV3-HD, 184.4; difference -0.10 (95% CI, -0.20, -0.00); Tables 2 and 3). A difference was observed in the rate of seroconversion between the treatment groups in the 2018–2019 season (aIIV3, 26.0% vs. HD-IIV3, 35.2%; difference -9.16 (95% CI -17.78, -0.54)) whereas there was no difference between the groups in the 2017–2018 season (aIIV3, 44.7% vs. HD-IIV3, 44.3%; difference 0.45 (95% CI -11.99, 12.90); Table 2). Reverse cumulative distribution curves summarizing participants HAI titer results from both seasons for influenza H3N2, H1N1 and B for each treatment group are shown in Supplementary Fig. 1.

**Table 3:** Hemagglutination Inhibition (HAI) antibody titers after trivalent adjuvanted inactivated influenza vaccine (aIIV3) and trivalent high-dose inactivated influenza vaccine (HD-IIV3) for each influenza vaccine strain by age for 2017–2018 and 2018–2019 influenza seasons

65–79 Years									
			aIIV3 Group			HD-IIV3 Group			aIIV3–HD-IIV3
Strain		Time	N	Value	95% CI	N	Value	95% CI	Difference 95% CI
H1N1	GMT^a^	Day 1^d^	269	52.5	(44.3, 62.2)	261	49.5	(41.4, 59.0)	0.03 (-0.08, 0.13)
		Day 29	269	108.8	(92.1, 128.5)	261	100.9	(84.9, 119.9)	0.03 (-0.07, 0.14)
	%SP^b^	Day 1	188	69.9	(64.0, 75.3)	171	65.5	(59.4, 71.2)	4.37 (-3.59, 12.33)
		Day 29	225	83.6	(78.7, 87.8)	216	82.8	(77.6, 87.1)	0.88 (-5.48, 7.25)
	%SC^c^	Day 29	80	29.7	(24.3, 35.6)	71	27.2	(21.9, 33.0)	2.54 (-5.14, 10.22)
H3N2	GMT	VDay 1	269	63.3	(51.7, 77.5)	261	56.4	(45.5, 69.8)	0.05 (-0.08, 0.18)
		Day 29	269	145.3	(124.3, 169.7)	261	184.4	(155.2, 219.1)	-0.10 (-0.20, -0.00)
	%SP	Day 1	181	67.3	(61.3, 72.8)	171	65.5	(59.4, 71.2)	1.77 (-6.27, 9.81)
		Day 29	246	91.4	(87.4, 94.4)	230	88.1	(83.6, 91.7)	3.33 (-1.83, 8.48)
	%SC	Day 29	92	34.2	(28.5, 40.2)	103	39.5	(33.5, 45.7)	-5.26 (-13.47, 2.94)
Influenza B	GMT	Day 1	269	12.2	(10.8, 13.8)	261	11.8	(10.3, 13.4)	0.02 (-0.06, 0.09)
		Day 29	269	20.3	(17.2, 24.1)	261	23.8	(20.0, 28.4)	-0.07 (-0.17, 0.04)
	%SP	Day 1	60	22.3	(17.5, 27.8)	52	19.9	(15.3, 25.3)	2.38 (-4.56, 9.33)
		Day 29	114	42.4	(36.4, 48.5)	118	45.2	(39.1, 51.5)	-2.83 (-11.28, 5.61)
	%SC	Day 29	51	19.0	(14.5, 24.2)	68	26.1	(20.8, 31.8)	-7.09 (-14.19, -0.00)
80 years and older									
H1N1	GMT^a^	Day 1^d^	73	49.3	(35.1, 69.3)	77	55.6	(39.2, 78.8)	-0.05 (-0.26, 0.16)
		Day 29	73	73.4	(55.2, 97.7)	77	88.7	(66.7, 118.0)	-0.08 (-0.25, 0.09)
	%SP^b^	Day 1	50	68.5	(56.6, 78.7)	55	71.4	(60.0, 81.0)	-2.94 (-17.61, 11.74)
		Day 29	60	82.2	(71.5, 89.9)	66	85.7	(75.9, 92.4)	-3.52 (-15.27, 8.23)
	%SC^c^	Day 29	16	21.9	(13.1, 33.1)	19	24.7	(15.6, 35.8)	-2.76 (-16.28, 10.76)
H3N2	GMT	Day 1	73	63.7	(41.4, 98.0)	77	66.5	(43.9, 101)	-0.02 (-0.27, 0.24)
		Day 29	73	129.2	(93.4, 178.8)	77	155.0	(114.2, 210.5)	-0.08 (-0.27, 0.11)
	%SP	Day 1	47	64.4	(52.3, 75.1)	51	66.2	(54.6, 76.5)	-1.85 (-17.09, 13.39)
		Day 29	61	83.6	(73.0, 91.0)	69	89.6	(80.6, 95.2)	-6.05 (-16.95, 4.85)
	%SC	Day 29	20	27.4	(17.6, 39.1)	27	35.1	(24.5, 46.7)	-7.67 (-22.44, 7.11)
Influenza B	GMT	Day 1	73	18.7	(14.6, 24.0)	77	31.2	(23.2, 42.1)	-0.22 (-0.39, -0.05)
		Day 29	73	27.0	(19.0, 38.2)	77	45.0	(31.8, 63.5)	-0.22 (-0.43, -0.01)
	%SP	Day 1	24	32.9	(22.3, 44.8)	39	50.6	(39.0, 62.2)	-17.77 (-33.29, -2.25)
		Day 29	37	50.7	(38.7, 62.5)	47	61.0	(49.2, 71.8)	-10.35 (-26.17, 5.46)
	%SC	Day 29	13	17.8	(9.8, 28.5)	11	14.3	(7.4, 24.1)	3.52 (-8.23, 15.27)

^a^ GMT: Geometric mean titers

^b^ % SP: Percent Seropositive, defined as HAI titer ≥ 1:40

^c^ % SC: Percent Seroconversion, defined as an HAI titer ≥ 1:40 at day 29 post-vaccination if the baseline pre-vaccination titer is < 1:10 or a minimum four-fold rise in HAI titer if the baseline pre-vaccination titer is ≥ 1:10

^d^ Day 1: Baseline blood draw before vaccination; Day 29 blood draw on 29 ± 7 days post-vaccination

For the A(H1N1)pdm09 and B strains there were no significant differences in percent seroconversion, Day 29 GMTs, and percent seropositive between the aIIV3 and HD-IIV3 groups for all ages (Table 2) or by age groups 65 to 79 years and 80 years and older, with two exceptions for the B strain (Table 3). The seroconversion for the B strain was significantly higher after HD-IIV-3 vs. aIIV3-HD in the 65–79 age group and the Day 29 GMT in the ≥ 80 age group was significantly higher after HD-IIV3 vs. aIIV3.

### Exploratory outcomes

**Fig. 2:**
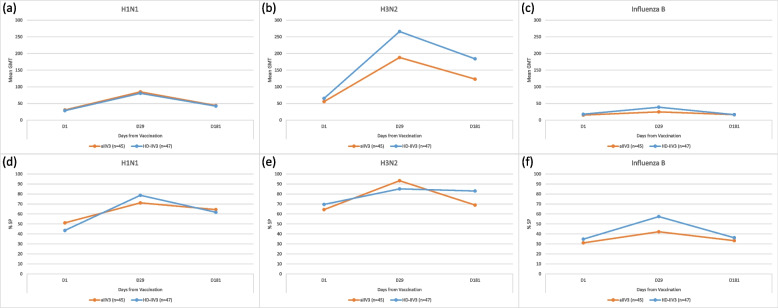
Mean hemagglutinin inhibition (HAI) geometric mean titers (GMT) and percent seropositive (% SP) (HAI titer ≥ 1:40) in the adjuvanted inactivated influenza (aIIV3) (n = 45) and high-dose inactivated influenza (HD-IIV3) (n = 47) groups for H1N1, H3N2 and B strains at Day 1 pre-vaccination and Day 29 and Day 181 post-vaccination

We found no relationship between HAI titer and at least one moderate/severe reactogenicity event (n = 146, 19.9%) for allV3 and HD-IIV3 treatment groups. There were statistically significant associations for sex and ethnicity for H1N1; treatment group, site, and year of study for H3N2; and sex, age, and year of study for influenza B and HAI titers (Table 4). For the subset of participants with serum collection 6 months post-vaccination, Fig. 2 shows changes in HAI GMT and percent seropositive at 29 days and 181 days after aIIV3 (n = 45) and HD-IIV3 (n = 47). There were no statistically significant differences between vaccine groups for any strain at 29 and 181 days post-vaccination at the 0.05 alpha level. Supplement Table 2 shows HAI GMT, seropositivity (SP) and seroconversion (SC) rates after aIIV3 and HD-IIV3 for each influenza vaccine strain at Day 1 (before vaccination) and Day 29 and Day 181 post-vaccination.

**Table 4:** Significant associations between hemagglutination Inhibition (HAI) titers and demographic measures (age, gender, race, ethnicity), site, treatment group, year of study, and statin use assessed using a linear regression model^a^

Influenza Vaccine Strain	Measure	Median Titer	GMT^b^	*p*-value
H1N1	Sex			0.0245
	Male	80	100.9	
	Female	160	127.3	
	Ethnicity			0.0196
	Hispanic	96.6	109.1	
	Non-Hispanic	80	85.4	
	Other	160	156.4	
H3N2	Treatment Group			0.0145
	allV3	160	257.8	
	HD-IIV3	160	326.5	
	Site			0.0010
	Boston Medical Center	160	226.1	
	Cincinnati Children’s Hospital	160	446.4	
	Duke University	160	241.9	
	Year of Study			< 0.0001
	2017–2018	320	452.2	
	2018–2019	160	186.1	
Influenza B	Gender			0.0025
	Male	40	28.9	
	Female	20	20.8	
	Age Group			0.0001
	65–79	20	18.7	
	80 +	40	32.2	
	Year of Study			< 0.0001
	2017–2018	40	32.6	
	2018–2019	20	18.5	

^a^Any of the independent variables (age, gender, race, ethnicity), site, treatment group, year of study, and statin use not presented per influenza strain were not significant in these regression analyses

^b^The GMTs for each outcome measure are adjusted in the model based on the other covariates

## Discussion

This is the first randomized clinical trial in the United States directly comparing immunogenicity following vaccination with aIIV3 or HD-IIV3 in older adults. For the primary outcome, we hypothesized that the seroconversion rate for the A(H3N2) strain after aIIV3 would be noninferior to HD-IIV3. We found that the proportion of participants that seroconverted at day 29 after vaccination after allV3 (32.8%) was statistically inferior to the proportion of participants that seroconverted after HD-IIV3 (38.4%) using a 10% noninferiority margin. The clinical significance of this finding is unclear. The difference in proportions (5.8%) between the groups was small. The difference in the rate of seroconversion between groups occurred only for the 2018–2019 season and not for the 2017–2018 season. The seropositive rates at day 29 post-vaccination for A(H3N2) were very high in both groups (aIIV3, 89.8%; HD-IIV3, 88.5%). There were no significant differences observed in GMTs at 29 days after vaccination in treatment groups after H1N1 or after B in the older age group; GMTs were significantly higher after HD-IIV3 vs. aIIV3 in the 65–79 age group. Furthermore, the proportion of subjects achieving seroconversion, for the H1N1 and influenza B vaccine strains showed no significant differences between the aIIV3 and HD-IIV3 vaccinated groups.

In a post licensure study in Hong Kong conducted during the 2017–2018 influenza season, Cowling et al. compared immune responses to aIIV3 (*n* = 200), HD-IIV3 (*n* = 200), and trivalent recombinant influenza vaccine (RIV3) (*n* = 200) with SD-IIV4 (*n* = 200) in community dwelling adults aged 65–82 years [2]. Authors observed no significant differences between aIIV3 and HD-IIV3 groups in HAI GMTs, mean fold rise, or percent with ≥ fourfold rise at day 30 post-vaccination for any vaccine strain. The proportion of participants with an HAI titer ≥ 1:40 at 30 days was 96% in the aIIV3 group and 96% in the HD-IIV3 group.

We explored whether a more robust immune response to either influenza vaccine was associated with increased reactogenicity. As there were few grade 3 (severe) reactions, analysis of the relationship between severe reactions and immune response was not possible. Instead, we combined moderate and severe reactions (19.9% of participants) and found no association between the magnitude of serologic immune responses and the occurrence of at least one moderate/severe reactogenicity event for either of the treatment groups. In a study of adjuvanted H1N1 influenza vaccine in 178 heathy participants aged 18–65 years old, 23% of individuals developed moderate-severe adverse events (local or systemic) [15]. The study found no correlation between these adverse events and serological vaccine response as measured by HAI and microneutralization assays. Thus, the data suggest that the severity of local or systemic reactions is not associated with the magnitude of the serologic immune response to influenza vaccine, but the topic deserves further study.

After adjusting for site, age group, treatment group, sex, race, and ethnicity our models found no effect of current statin use on HAI titers compared to no use of statins. This finding differs from that of a cross-sectional observational study of statin therapy on the immune response to influenza vaccine in 6961 subjects aged 65 years and older, which was nested within a comparative immunogenicity clinical trial of adjuvanted versus unadjuvanted influenza vaccine [1]. In that study, HAI GMTs to influenza A(H1N1)pdm09, A(H3N2), and B strains were significantly lower at 22 days post-vaccination in subjects receiving statin therapy, defined as taking statins from ≥ 28 days before through 22 days after vaccination, compared with those not receiving statin therapy. However, this finding was limited by using self-report for statin use and lack of detail on statin dose and duration of therapy.

In a subset of participants assessed for waning immunity 6 months after vaccination in season 1, the proportion of participants with seropositivity remained higher 6 months after vaccination than at baseline for H3N2 and H1N1 A strains; > 60% of participants had seropositivity at 6 months post-vaccination for these strains. The mean GMTs for the A strains were also higher at 6 months vs at baseline. Immunogenicity was lower for the B strains for both vaccines, and at 6 months the proportion of participants with seropositivity and mean GMTs were similar to baseline.

This study is subject to some limitations. The study population was drawn from a population of community-dwelling older adults, who were generally healthy, mostly white, and well educated (> 85% had some college education or more), and therefore not representative of less well, frail, non-white, lower-educated older adults. The study included only two influenza seasons. There were no measures of cellular immunity. Although we used standard serological measures of immune response to influenza vaccine, antibody responses appear to be less reliable measures of protection in older adults with multiple co-morbidities or frailty compared to measures of cellular immunity [13]. HAI titers are also not a direct measure of influenza vaccine effectiveness. Although HAI titers may be a correlate of protection for influenza illness in older adults, individual post vaccination titers do not reliably classify a person as protected [4, 17].

## Conclusion

In our trial comparing aIIV3 and HD-IIV3 in older adults, overall immunogenicity findings were similar after aIIV3 and HD-IIV3. For the primary outcome of seroconversion for H3N2, the aIIV3 seroconversion rate was inferior to HD-IIV3, but the HD-IIV3 seroconversion rate was not statistically superior to the aIIV3 seroconversion rate.

## Methods

### Study design and population

We conducted a prospective, randomized, blinded clinical trial at CDC-sponsored Clinical Immunization Safety Assessment (CISA) Project sites during the 2017–2018 (Duke University Medical Center and Boston Medical Center) and 2018–2019 (Duke, Boston, and Cincinnati Children’s Hospital Medical Center) influenza seasons. The study protocol was approved by institutional review boards at each study site and was registered at ClinicalTrials.gov (NCT03183908); CDC relied on the Duke institutional review board. We previously described design and safety outcomes from this study [14]. In brief, the eligibility criteria included age ≥ 65 years, community-dwelling, not immunosuppressed, cognitively intact, able to speak English, and no contraindications to influenza vaccination [14]. We aimed to have at least 20% of the study population to be ≥ 80 years old.

After obtaining written informed consent on Day 1, staff collected demographic, medical history, and medication information on each eligible participant. Participants had blood drawn for antibody studies on Day 1 (vaccination day) before vaccination and on Day 29 ± 7 days post-vaccination. At the Duke site only, a convenience sample of participants had blood drawn on Day 181 ± 14 days in year one. At enrollment, participants were randomized (1:1) to receive aIIV3 or HD-IIV3 using a permuted block randomization scheme stratified by study site. Separate permuted blocks were used for participants aged 65 − 79 and ≥ 80 years. Participants and study staff performing data collection and analysis were blinded to treatment allocation.

Following randomization, a 0.5 mL intramuscular dose of either U.S.-licensed aIIV3 or HD-IIV3 was administered in the deltoid muscle by a designated unblinded vaccinator, who did not participate in other aspects of the study. Each aIIV3 dose contained 15 mcg of hemagglutinin (HA) from each of the three recommended influenza strains for the respective season and MF59 adjuvant, a squalene-based, oil-in-water emulsion. Each HD-IIV3 dose contained 60 mcg of HA from each of the three recommended influenza strains for the respective season (Supplement Table 1) [14].

### Laboratory methods

#### Influenza hemagglutination inhibition (HAI) assay

Reference vaccine virus strains (Supplement Table 1) representative of the specific viral antigens included in the 2017–2018 and 2018–2019 influenza vaccines and propagated in embryonated chicken eggs were used to evaluate the relative levels of all three influenza strain-specific antibodies in participant serum samples. The HAI assay was performed in accordance with the Duke Regional Biocontainment Laboratory Virology Unit’s fully optimized and approved protocol (RVUSOP004 Influenza HI of Serum Samples). Test samples were assayed by HAI as duplicate twofold dilution series starting at 1:10. Serum dilutions are then incubated with a concentration of virus verified to possess a known potential for red blood cell (RBC) agglutination. The presence of virus-specific antibodies was visualized via incubation of the virus-serum mixture with RBC solution; the endpoint titer for a given dilution series was then expressed as the reciprocal of the final dilution in which complete HAI is observed. By convention, seronegative samples are defined as having an endpoint HAI titer < 40. Seropositive samples are defined as having an endpoint titer of ≥ 1:40. Seroconversion is defined as an HAI titer ≥ 1:40 at day 29 post-vaccination if the baseline pre-vaccination titer is < 1:10 or a minimum four-fold rise in HAI titer if the baseline pre-vaccination titer is ≥ 1:10 [11].

### Outcome measures

The primary outcome was the proportion of subjects achieving A(H3N2) seroconversion at day 29 for the respective season’s vaccine component.

Secondary outcomes included the proportion of subjects achieving seroconversion at day 29 for A(H1N1)pdm09 or B strains, proportions of subjects in each age group achieving seroprotection at day 29 for A(H3N2), A(H1N1)pdm09 or B strains, proportions of subjects achieving post-vaccination HAI titer ≥ 1:40 for each season’s vaccine strains, and post-vaccination HAI geometric mean titers (GMT). Exploratory outcomes included associations between HAI titers and moderate/severe local and systemic reactogenicity events as the dependent variable [14]. Exploratory outcomes also included associations between age, sex, race, ethnicity, and statin use and HAI titers as the dependent variable. We assessed changes in HAI titers from baseline and at 181 days post-vaccination in a subset of subjects at the Duke study site only during the 2017–2018 season study year.

### Statistical analysis

The planned sample size of at least 780 evaluable participants (390 per group across all sites).provided approximately 80% power to reject the null hypothesis that the proportion of participants that seroconverted at day 29 for the A(H3N2) strain after aIIV3 is inferior to HD-IIV3 assuming a 50% seroconversion rate. The statistical testing for the primary immunogenicity outcome was conducted at the 1-sided α = 0.025 level using the upper bound of a Newcombe binomial confidence interval [18] stratified by study site with Cochran-Mantel–Haenszel (CMH) weighting with a noninferiority margin of 10%. Comparisons of seropositivity and seroconversion rates were made using the immunogenicity population that consisted of all participants who were randomized, vaccinated, and provided baseline and day 29 post-immunization blood draws of acceptable volume and quality within the protocol-defined time frame with no protocol violations affecting immunogenicity.

The comparison of seropositivity and seroconversion rates (with the exception of the primary outcome above) between treatment groups was made using exact binomial 95% CIs. We calculated the 95% CI on the difference between mean proportions for seropositivity and seroconversion rates and log10 transformed GMTs between treatment groups (aIIV3 minus HD-IIV3); a difference that did not cross 0 was considered a statistically significant difference. The relationship between immunogenicity and reactogenicity for allV3 and HD-IIV3 was assessed using a logistic regression model with at least one moderate/severe reactogenicity event as the dependent variable and covariates of the post-vaccination strain-specific log10 HAI titer, site, age group and treatment group. The assessment of factors associated with immunogenicity for allV3 and HD-IIV3 was made with a linear regression model with the log10 HAI titer as the dependent variable with site, age group, treatment group, sex, ethnicity, race, year of study, and statin use as covariates. The comparison of seropositivity and seroconversion rates and changes in serum hemagglutination inhibition at one month and six months after vaccination were made using a Cochran-Mantel–Haenszel test. The exploratory objectives described above included participants who were randomized, vaccinated, and had complete immunologic data. We used a 2-sided α = 0.05 level for all the exploratory analyses with no adjustment for multiple comparisons. These data were analyzed using SAS statistical software version 9.4 (SAS Institute).

## Supplementary Information

**Additional file 1: Supplementary Figure 1.** Reverse cumulative distribution curves summarizing participants HAI titer results from both seasons for influenza H3N2, H1N1 and B for each treatment group.

**Additional file 2: Supplementary Table 1. **Virus strains in the trivalent adjuvanted inactivated influenza vaccine (aIIV3), the trivalent high dose inactivated influenza vaccine (HD-IIV3) and the influenza hemagglutination inhibition assay test viruses, 2017-2018 and 2018-2019 influenza seasons.** Supplementary Table 2.**Hemagglutination Inhibition (HAI) geometric mean antibody titers (GMT) and sero-protection (SP) and seroconversion (SC) rates after aIIV3 (*n*=45) and HD-IIV3 (*n*=47) for each influenza vaccine strain at Day 1 (before vaccination) and Day 29 and Day 181 post-vaccination for 2017-2018 influenza season.

## Data Availability

Individual deidentified participant data will not be shared.
