# Supplementary material for: Immunogenicity of adjuvanted versus high-dose inactivated influenza vaccines in older adults: a randomized clinical trial
Source: Immun Ageing. 2023 Jul 1;20:30. doi: 10.1186/s12979-023-00355-7 (PMC10314373; doi:10.1186/s12979-023-00355-7)
Supplement: Supplementary file 2 — Additional file 2: Supplementary Table 1. Virus strains in the trivalent adjuvanted inactivated influenza vaccine (aIIV3), the trivalent high dose inactivated influenza vaccine (HD-IIV3) and the influenza hemagglutination inhibition assay test viruses, 2017-2018 and 2018-2019 influenza seasons. Supplementary Table 2.Hemagglutination Inhibition (HAI) geometric mean antibody titers (GMT) and sero-protection (SP) and seroconversion (SC) rates after aIIV3 (n=45) and HD-IIV3 (n=47) for each influenza vaccine strain at Day 1 (before vaccination) and Day 29 and Day 181 post-vaccination for 2017-2018 influenza season. [file 12979_2023_355_MOESM2_ESM.docx]

Supplementary Table 1. Virus strains in the trivalent adjuvanted inactivated influenza vaccine (aIIV3), the trivalent high dose inactivated influenza vaccine (HD-IIV3) and the influenza hemagglutination inhibition assay test viruses, 2017-2018 and 2018-2019 influenza seasons.

| Influenza Vaccine Strain | 2017-18 | 2018-19 |
| --- | --- | --- |
| H1N1 | A/Michigan/45/2015 (H1N1) pdm09-like virus | A/Michigan/45/2015 (H1N1)pdm09-like virus |
| H3N2 | A/Hong Kong /4801/2014 (H3N2)-like virus | A/Singapore/INFIMH-16-0019/2016 A(H3N2)-like virus |
| Influenza B | B/Brisbane/60/2008-like virus (Victoria lineage) | B/Colorado/06/2017-like virus (Victoria lineage) |

Supplementary Table 2. Hemagglutination Inhibition (HAI) geometric mean antibody titers (GMT) and sero-protection (SP) and seroconversion (SC) rates after aIIV3 (n=45) and HD-IIV3 (n=47) for each influenza vaccine strain at Day 1 (before vaccination) and Day 29 and Day 181 post-vaccination for 2017-2018 influenza season.

|  |  |  |  | aIIV3 |  |  | HD-IIV3 |  |
| --- | --- | --- | --- | --- | --- | --- | --- | --- |
| Influenza Vaccine Strain | Measure | Day | n | Value | 95% CI | n | Value | 95% CI |
| H1N1 | GMT^1^ | 1 | 45 | 30.5 | 21.8, 42.7 | 47 | 28.5 | 21.2, 38.4 |
|  |  | 29 | 45 | 84.9 | 51.4, 140.0 | 47 | 80.6 | 51.9, 125.1 |
|  |  | 181 | 45 | 43.9 | 26.4, 73.0 | 47 | 42.1 | 25.9, 68.4 |
|  | %SP^2^ | 1 | 23 | 51.1 | 35.8, 66.1 | 20 | 43.5 | 28.9, 58.7 |
|  |  | 29 | 32 | 71.1 | 55.7, 83.3 | 37 | 78.7 | 64.3, 88.9 |
|  |  | 181 | 29 | 64.4 | 48.8, 77.9 | 29 | 61.7 | 46.4, 75.3 |
|  | %SC^3^ | 29 | 16 | 35.6 | 21.9, 51.1 | 19 | 41.3 | 27.0, 56.6 |
|  |  | 181 | 9 | 20.0 | 9.6, 34.5 | 9 | 19.6 | 9.4, 33.9 |
| H3N2 | GMT | 1 | 45 | 55.7 | 37.8, 82.0 | 47 | 65.3 | 44.0, 96.9 |
|  |  | 29 | 45 | 188.1 | 120.5, 293.6 | 47 | 266.1 | 160.8, 440.5 |
|  |  | 181 | 45 | 123.1 | 71.2, 212.9 | 47 | 184.1 | 109.0, 310.8 |
|  | %SP | 1 | 29 | 64.4 | 48.8, 77.9 | 32 | 69.6 | 54.2, 82.0 |
|  |  | 29 | 42 | 93.3 | 81.7, 98.2 | 40 | 85.1 | 71.7, 93.4 |
|  |  | 181 | 31 | 68.9 | 53.4, 81.5 | 36 | 83.0 | 69.2, 92.0 |
|  | %SC | 29 | 19 | 42.2 | 27.7, 57.7 | 23 | 50.0 | 34.9, 64.9 |
|  |  | 181 | 11 | 24.4 | 12.9, 39.5 | 16 | 34.8 | 21.4, 50.1 |
| B | GMT | 1 | 45 | 15.2 | 11.0, 20.9 | 47 | 17.7 | 12.6, 25.0 |
|  |  | 29 | 45 | 24.8 | 15.8, 39.0 | 47 | 38.8 | 25.2, 59.9 |
|  |  | 181 | 45 | 16.5 | 10.7, 25.4 | 47 | 16.5 | 10.5, 25.9 |
|  | %SP | 1 | 14 | 31.1 | 18.2, 46.5 | 16 | 34.8 | 21.4, 50.1 |
|  |  | 29 | 19 | 42.2 | 27.7, 57.7 | 27 | 57.4 | 42.2, 71.5 |
|  |  | 181 | 15 | 33.3 | 20.0, 48.8 | 17 | 36.2 | 22.7, 51.4 |
|  | %SC | 29 | 10 | 22.2 | 11.2, 37.0 | 13 | 28.3 | 16.0, 43.4 |
|  |  | 181 | 5 | 11.1 | 3.7, 24.0 | 0 | 0.0 | . , 7.7 |

| ^1^GMT | Geometric Mean Titer |
| --- | --- |
| ^2^%SP | Percent Seropositive |
| ^3^%SC | Percent Seroconversion |
